# Predicting prostate cancer recurrence using an atlas‐based tumor control probability model

**DOI:** 10.1002/mp.70282

**Published:** 2026-01-14

**Authors:** Kazi Ridita Mahtaba, Martin A. Ebert, Jeremy Booth, Leyla Moghaddasi, Robert Finnegan, Yutong Zhao, Burhan Javed, George Hruby, Annette Haworth

**Affiliations:** ^1^ Institute of Medical Physics, School of Physics The University of Sydney Camperdown New South Wales Australia; ^2^ School of Physics Mathematics and Computing, University of Western Australia Crawley Western Australia Australia; ^3^ Department of Radiation Oncology Sir Charles Gairdner Hospital Perth Western Australia Australia; ^4^ Centre for Advanced Technologies in Cancer Research Centre for Advanced Technologies in Cancer Research Perth Western Australia Australia; ^5^ Northern Sydney Cancer Centre Royal North Shore Hospital St Leonards New South Wales Australia; ^6^ Genesis Care Rockhampton Hospital Rockhampton Queensland Australia

**Keywords:** prostate cancer recurrence, radiosensitivity parameters, tumor control probability

## Abstract

**Background:**

Recurrence following prostate cancer (PCa) radiation therapy (RT) remains a persistent challenge. Although dose escalation can improve tumor control, it often results in increased toxicity. With an understanding of tumor heterogeneity, identification of radioresistant tumor subvolumes at risk of low tumor control probability (TCP) could provide an opportunity for personalized dose prescription to reduce risk of treatment failure without the increased risk of toxicity.

**Purpose:**

The aim of this study was to evaluate the efficacy of an atlas‐based tumor control probability model in predicting prostate cancer recurrence by retrospectively integrating patient‐specific primary radiotherapy and histopathology‐informed data. A segment‐wise adjustment to TCP model parameters was investigated for enhancing recurrence prediction in a patient cohort with biopsy‐confirmed local recurrence following definitive RT.

**Methods:**

Nine patients with biopsy proven local recurrence were selected from an ethics‐approved study (NCT03073278) based on the availability of histopathology reports, dose‐fractionation schedules and treatment planning data from their primary RT. Two previously‐reported population‐based biological atlases, one comprising a cell density data (CD‐atlas) and the other tumor probability data (TP‐atlas), were deformably registered to the prostate contour of each patient. Histopathology reports were retrieved for each patient, and the registered prostate atlases were anatomically segmented based on individual histopathology findings. Radiosensitivity parameters were derived from a separate patient cohort's histology dataset using a numerical optimization method, generating a single PCa grade‐independent α/β ratio, four Gleason Pattern (GP)‐dependent α parameters, and nine Gleason Score (GS)‐dependent α/β ratios. Three parameter adjustment approaches—cell density alone, cell density with GP‐dependent α, and cell density with GS‐dependent α/β, were evaluated and compared to a baseline model without adjustments. Changes in overall TCP values resulting from the adjustments were analyzed, and recurrent gross tumor volume (GTV) contours were overlaid on the TCP maps to evaluate their alignment with regions of lower TCP, assessing the model's ability to enhance recurrence prediction.

**Results:**

The approach combining segment‐wise cell density and GS‐dependent α/β adjustments showed superior predictive capability, with all nine patients (100%) exhibiting a significant (*p* = 0.004) reduction in overall TCP values and seven patients (78%) showing alignment of lower TCP regions with relapsed tumor sites. This was further supported by voxel‐level histogram analysis and statistically significant volume‐weighted TCP differences between GTV and nonGTV regions (Wilcoxon signed‐rank test, *p* = 0.003). In contrast, GP‐dependent α adjustments alongside cell density failed to predict recurrence, while cell density adjustments alone yielded moderate performance. Additionally, the generated single α/β ratio and GS‐dependent α/β ratios were consistent with the lower α/β ratios typically associated with PCa.

**Conclusions:**

The atlas‐based TCP model, enhanced with patient‐specific histopathology report data, demonstrated promising capabilities in predicting PCa recurrence. This approach has the potential to support personalized treatment planning by enabling optimization of the distribution of a specific integral dose to minimize recurrence risk.

## 1 INTRODUCTION

Radiotherapy is an effective treatment modality for localized prostate cancer (PCa), yet recurrence after radiation remains a critical issue for a subset of patients.^1^, ^2^ Local recurrences often originate from the primary tumor site,^3^ suggesting that focal dose escalation to the tumor, while maintaining standard doses for the rest of the prostate, could improve outcomes.^4^, ^5^ The FLAME trial demonstrated that such targeted dose escalation significantly enhances 5‐year biochemical disease‐free survival, local control, and reduction in distant metastases without increasing toxicity.^3^, ^4^Beyond dose escalation, understanding tumor biology can further improve treatment strategies. The Gleason Score (GS) is a grading system for prostate tumors, determined by summing the two most predominant Gleason Patterns (GP) observed in the diagnostic biopsy sample. A higher score indicates a more aggressive tumor with increasingly disorganized and irregular cellular pattern.^6^ Developing predictive tools that integrate spatial tumor characteristics within intraprostatic lesions may be essential for guiding targeted dose escalation and achieving better disease control with minimal toxicity.

Rather than simply boosting the MRI or PSMA PET evident lesion, a biologically targeted radiation therapy (BiRT) approach is the focus of our research group, which aims to integrate image‐derived, voxel‐level tumor characteristics into the treatment planning process.^7^, ^8^, ^9^, ^10^ This method utilizes a tumor control probability (TCP) model which combines established linear‐quadratic (LQ) TCP models to address variability in spatial dose, dose rate and tumor characteristics.^11^ Our team has previously demonstrated, through in silico studies, methods to incorporate patient‐specific, voxel‐level information including selected radiobiological parameters in fractionated external beam radiotherapy (EBRT) and brachytherapy treatments.^7^, ^9^, ^10^, ^12^, ^13^, ^14^ The voxel‐wise TCP dose‐optimization model seeks to maximize tumor control probability for individual voxels while minimizing the risk of complications in surrounding healthy tissue. Historically, biological dose‐optimization techniques using TCP models often rely on generic, previously published parameter sets, overlooking patient‐specific tumor characteristics such as heterogeneous clonogenic cell distributions. This approach may lead to misinterpretation of tumor control probability, increasing the risk of treatment failure. Tumor location and characteristics can be derived from machine learning estimates based on multi‐parametric MRI (mpMRI).^12^, ^15^ However, the accuracy of mpMRI‐based predictions is limited by inconsistencies in scanning parameters relative to those used in predictive model development.^12^ To address this, Finnegan et al.^16^ developed a statistical biological model (biological atlas) that summarizes voxel‐level, three‐dimensional (3D) histology data distributions from a cohort of 63 patients, using probability distributions to represent the histological features. Previous work by Zhao et al.^7^ developed Gleason Pattern (GP)‐specific radiosensitivity parameters and in a later work^12^ applied Finnegan et al.'s^16^ cell density (CD) and tumor probability (TP) atlas. In another study, Haworth et al.^17^ mathematically divided the prostate into 12 subsections and adjusted tumor cell densities using data from patient biopsy reports, aiming to enhance the predictive accuracy of the TCP model. The biological atlas can serve as a foundation for TCP modeling and can be further refined by incorporating patient‐specific information from histopathology reports, with the potential to strengthen TCP predictions tailored to individual patients.

We hypothesize that adjusting model parameters to reflect tumor grading and clonogenic cell density based on histopathology could improve recurrence prediction by producing more realistic TCP values. Moreover, a map visualizing the voxel‐wise TCP distribution could help identify regions with lower TCP values, potentially indicating areas at higher risk of recurrence. Our study aimed to establish voxel level tumor grade (Gleason Score or Gleason Pattern)‐dependent radiosensitivity parameters and incorporate these into the atlas‐based TCP model for a cohort of prostate cancer patients with documented local recurrence. By retrospectively integrating tumor characteristics from histopathology reports, we aimed to enhance our model's ability to predict recurrence both quantitatively and qualitatively which respectively involved calculating overall TCP and generating voxel‐wise TCP maps to localize regions at higher risk of recurrence. This work is therefore presented as a proof‐of‐principle study, demonstrating the feasibility of incorporating histopathology‐informed parameters into an atlas‐based TCP model for recurrence prediction.

## 2 METHODS

**FIGURE 1:**
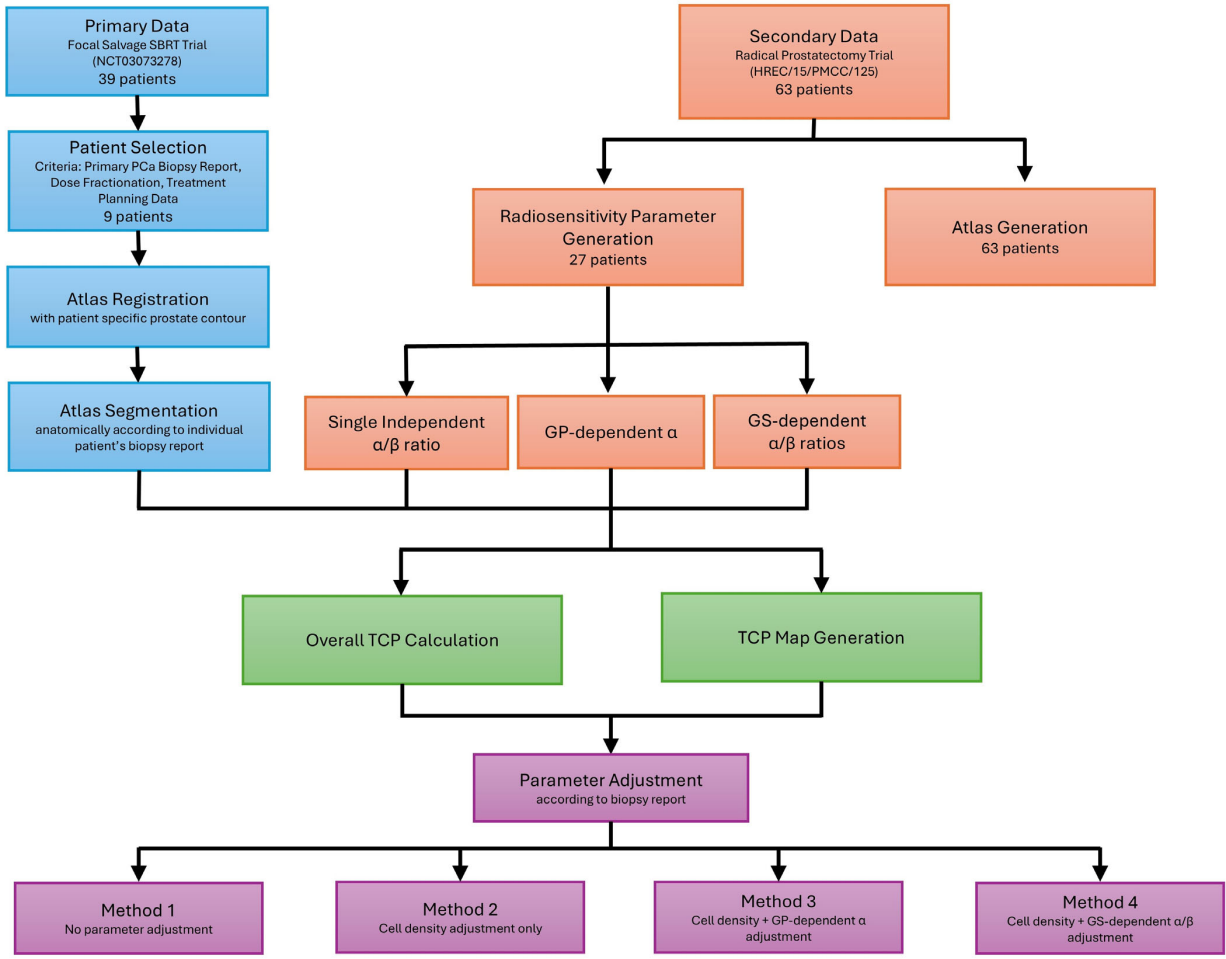
A flowchart displaying various steps from the methodology to calculate overall TCP and generate TCP distribution maps for the analysis.

This study followed a multi‐stage analysis pipeline, beginning with data acquisition and atlas registration, followed by histopathology report‐based segmentation, radiosensitivity parameter generation, TCP modelling, and comparative evaluation, which is summarized in the flowchart shown in Figure 1. In this work, histopathology data were used solely to inform the physics‐based TCP modelling process, providing ground‐truth information on tumor location and aggressiveness, and were not analyzed for primary pathology research.

### 2.1 Data acquisition

#### 2.1.1 Primary data

Patient data were derived from a prospective focal stereotactic body radiation therapy (SBRT) re‐irradiation study (NCT03073278), approved by the Human Research Ethics Committee at the Royal North Shore Hospital in Sydney, Australia.^1^ Eligibility criteria included biopsy‐confirmed localized prostate cancer recurrence following definitive irradiation, concordant findings on multiparametric MRI and ^68^Ga‐prostate‐specific membrane antigen (PSMA) positron emission tomography/computed tomography (PET/CT), and a prostate‐specific antigen (PSA) level below 15 ng/mL at the time of recurrence. Initially, five patients were selected from a cohort of 39 based on the availability of primary cancer histopathology (biopsy) reports, DICOM (Digital Imaging and Communications in Medicine) treatment planning data, and primary radiotherapy dose‐fractionation schedules recorded in the hospital's oncology information system. Subsequently, four additional patients were included who met all selection criteria except for the availability of primary DICOM data; however, DICOM data from their re‐treatment was available and used instead. A summary of this cohort's demographics is provided in Table S‐1 (Supporting Information).

#### 2.1.2 Secondary data and atlas generation

For generating radiosensitivity parameters (Section 2.5), data from prostate cancer patients from an independent Human Research Ethics Committee‐approved project (HREC/15/PMCC/125,Peter MacCallum Cancer Centre, Melbourne, Australia), was accessed.^7^, ^12^, ^16^ The original study comprised 63 patients with fully annotated, whole‐mount histology data from radical prostatectomy specimens. Radiosensitivity parameters were generated using an optimization method from a cohort of 27 randomly selected patients as described by Zhao et al.^7^ This study specifically utilized tumor cell number data, categorized by Gleason Score (GS) and Gleason Pattern (GP), derived from the histology data of those patients. The biological atlases, developed by Finnegan et al.,^16^ were created using the histology data from all 63 patients. Histopathology images and in vivo prostate MRI images from this radical‐prostatectomy patients’ cohort were coregistered to generate three‐dimensional maps of tumor grade distributions and cell density. These data were mapped to a common reference geometry, enabling the creation of voxelized statistical models resulting in two biological atlases: a cell density atlas (CD‐atlas) and a tumor probability atlas (TP‐atlas).^16^

### 2.2 Atlas registration

The CD‐atlas and TP‐atlas were deformably registered^16^ to the prostate contour of each of the nine patients in the primary data cohort. For the first five patients, DICOM data from their primary treatment stage were accessible in the treatment planning system, allowing the atlases to be registered with their prostate contours from the initial cancer treatment phase. For the remaining four patients, only DICOM data from the salvage treatment stage were available. After verifying through past medical reports that prostate volume had changed by no more than ±1.5 mL at the time of salvage SBRT, the atlases were registered to the salvage treatment prostate contour. To evaluate the accuracy of this registration process, the mean absolute surface distance (MASD) between atlas and patient prostate contours was calculated, which was consistently <1.5 mm across all nine patients. Each registered atlas was then treated as the patient‐specific prostate anatomy onto which our TCP model was applied.

### 2.3 Histopathology report and atlas segmentation

**FIGURE 2:**
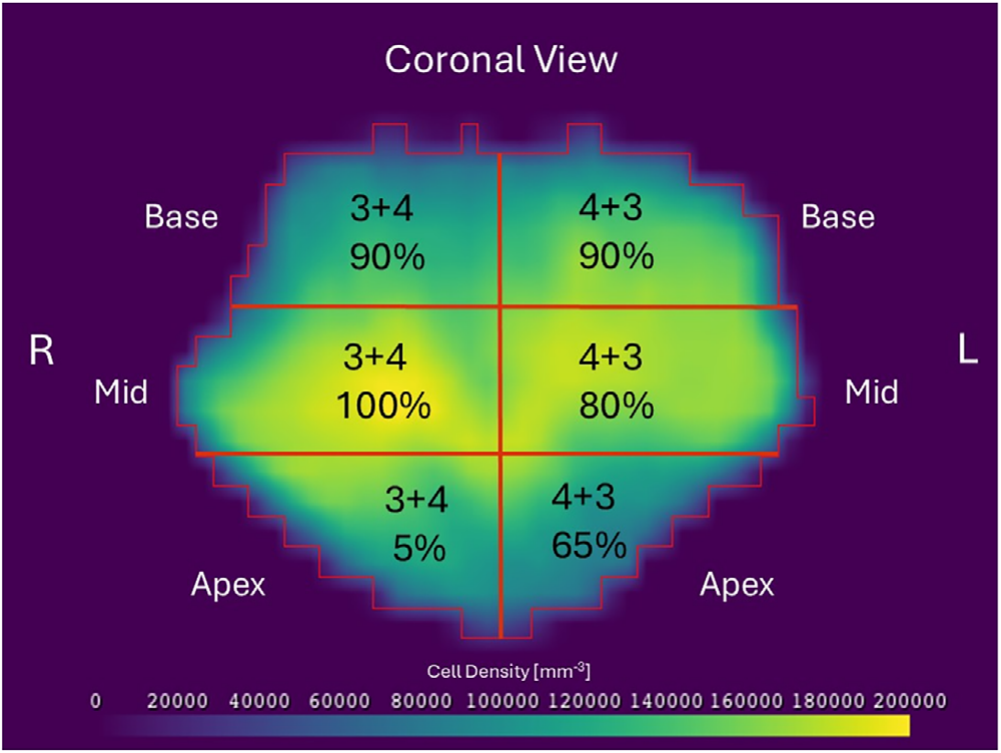
A diagram of prostate cell density atlas divided into six segments (right base, right mid, right apex, left base, left mid, and left apex) following the biopsy report of Patient‐1. Each segment is assigned with a Gleason Score and percentage of cancerous cells found in the core biopsy. The color scale represents cell density, measured as the number of cells per unit voxel volume.

Histopathology reports for all nine patients were retrieved from the hospital information system (ARIA, Version 15.6, Varian Medical Systems). Biopsy sample collection and reporting methods varied across patients, with each report dividing the prostate into six to twelve anatomical segments (e.g., left apex, right base, mid‐gland etc.), from which needle core samples were collected. For segments with detected cancerous cells, Gleason Scores and the percentage of adenocarcinoma (a type of cancer that starts in the glands) involved in each core were reported. To align with this reporting format, each patient's registered prostate atlas was divided into anatomical segments matching the biopsy sampling regions, using Python (version 3.11.7) scripts and the individual biopsy data. This segmentation provided a method to adapt the TCP model for each segment according to the specific histopathology findings. Figure 2 illustrates a two‐dimensional schematic representation of prostate atlas segmentation and assignment of GS and percentage of adenocarcinoma (in core samples) based on the histopathology report of Patient‐1.

### 2.4 TCP model: Overall TCP calculation and map generation

The voxel‐wise TCP model used in this study is an enhanced version of the linear‐quadratic TCP model, previously detailed in studies by Her et al.^9^, ^10^, ^18^ and Zhao et al.^7^, ^12^ This model calculates TCP on a voxel‐by‐voxel basis, where the equation incorporates parameters such as tumor cell count N_i_, a log‐normal distribution of radiosensitivity parameters α and α/β ratio, dose per fraction *d*_i_, total number of fractions *n*, total treatment duration *T*_exp_, and potential doubling time *T*_pot_. The general TCP model is given by the following equation:

(1) TCPiNi,M,αk,di=expαkndi2αβ−Ni,Mexp×−αkndi−αkndi2αβ+ln(2)TexpTpotαkε[0.05,0.4]

Here *α*_k_ is sampled from a log‐normal distribution in the range [0.05, 0.40] with mean $\bar{\alpha }$ = 0.15 Gy^−1^and standard deviation *σ*_α _= 0.04 Gy^−1^.^7^, ^9^, ^12^, ^19^ Based on this general formulation, the overall TCP for the whole prostate was calculated and voxel‐level TCP maps were generated for all nine patients. For the first five patients, actual dose distributions from the available treatment planning data were used in the TCP equation. However, for the last four patients, treatment planning data was unavailable in the system, so a uniform dose distribution was assumed based on the known dose prescription. A detailed description of the TCP model equations, overall TCP calculation, and map generation methods is provided in the Supporting Information, Section 2.

### 2.5 Radiosensitivity parameter generation

To support our hypothesis, it was essential to generate Gleason grade‐dependent radiosensitivity parameters. Previously, Zhao et al.^7^ derived four Gleason Pattern (GP)‐specific α parameters across a range of α/β ratios, utilizing the secondary data described in Section 2.1.2. Their approach involved setting TCP objectives based on outcomes of biochemical or clinical failure‐free survival at 5 years for several patient cohorts receiving three different dose‐fractionation schedules in the “Conventional or Hypofractionated High Dose Intensity Modulated Radiotherapy for Prostate Cancer” or CHHiP trial.^20^ Using the differential evolution (DE) algorithm^21^ with 500 iterated optimizations steps, they conducted numerical searches across a range of α/β ratios to avoid local minima and determine optimal *α*_GP_ ​values. In this study, we employed the same dataset and DE code (SciPy library, version 1.11.4 in Python) for generating GP‐specific *α* values, and with minor code adjustments to produce GS‐specific α/β ratios. For further details on the methodology, refer to Zhao et al.^7^

#### 2.5.1 Single α/β ratio

Initially, a single GS or GP independent α/β ratio was generated. At each optimization step of the DE algorithm, *α* was sampled from a log‐normal distribution with $\bar{\alpha }$ = 0.15 Gy^−1^and *σ*_α _= 0.04 Gy^−1^ within the range [0.05, 0.4]. The total tumor cell number *N*_i_ for each of the 27 patients (secondary data) was incorporated into Equation (1), while dose, fractionation number, and *T*_exp_ ​values were taken from the CHHiP trial data. The DE algorithm then optimized the average TCP across all 27 patients for each dose fraction schedule, aligning it with the corresponding TCP outcomes from the CHHiP trial to numerically solve for the optimal α/β ratio.

#### 2.5.2 GP‐dependent α

This process closely followed Zhao et al.'s^7^ method for generating Gleason Pattern dependent alpha parameters. Four GP‐dependent alpha (*α*_GPi_​) were generated based on the newly derived single α/β ratio and each patient's GP‐categorized tumor cell numbers N_GPi_​. It was assumed that less differentiated or more aggressive tumors exhibit lower radiosensitivity,^7^, ^22^ with *α* values ranked as follows:

$$\alpha_{GP2}>\alpha_{GP3}>\alpha_{GP4}>\alpha_{GP5}$$

#### 2.5.3 GS‐dependent α/β ratios

In this step, nine Gleason Score (2+2,3+2,3+3,3+4,4+3,4+4,4+5, 5+4, 5+5)‐dependent α/β ratios were generated similar to the method described for a single α/β ratio. However, instead of using each patient's total tumor cell number N_i_, the GS‐categorized cell numbers N_GSj_ ​were applied in the TCP equation. Since more aggressive tumors (higher GS) are expected to be more radioresistant, a lower TCP is anticipated for the same dose compared to less aggressive tumors. To establish the ranking relationship among GS‐dependent α/β ratios, a sensitivity analysis was conducted between TCP and α/β ratios, using fixed parameters (N_i_​, d_i_​, n, etc.). The analysis revealed a consistent decrease in TCP with increasing α/β ratios, exhibiting a nearly linear trend beyond α/β = 1.5 Gy (Figure S1). Based on this analysis, the following ranking condition was incorporated into the DE optimization algorithm to generate the nine GS‐dependent α/β ratios (α/β_GSj_):

$$\begin{array}{cc} & \alpha /\beta_{GS2+2}<\;\alpha /\beta_{GS3+2}<\;\alpha /\beta_{GS3+3}<\;\alpha /\beta_{GS3+4}\\ & <\;\alpha /\beta_{GS4+3}<\;\alpha /\beta_{GS4+4}<\;\alpha /\beta_{GS4+5}\\ & <\;\alpha /\beta_{GS5+4}<\;\alpha /\beta_{GS5+5} \end{array}$$

Considering the well‐established evidence that prostate cancer has a low α/β ratio,^23^, ^24^, ^25^ the lower boundary for α/β_GSj_ was set at 1 Gy and the upper boundary was set at 8.3 Gy.^7^, ^20^

### 2.6 Parameter adjustment

To calculate overall TCP and generate TCP maps, segment‐wise parameter adjustments were made based on each patient's histopathology report using three different methods, in addition to a baseline model without any adjustments. Given that all patients experienced PCa recurrence, a reduction in overall TCP and lower TCP regions on the map—likely overlapping with the relapsed tumor areas—were expected after parameter adjustments, however, each method was investigated to determine the most reliable method of predicting the risk and location of tumor recurrence.

#### 2.6.1 Method 1: [No parameter adjustment]

No segment‐wise adjustments based on histopathology reports were applied. The generated single α/β ratio and log‐normal distribution for alpha were used across the entire prostate, and tumor cell numbers *N*_i_ were taken directly from the CD‐atlas. The overall TCP and map from this method served as the baseline for comparison with the other three methods.

#### 2.6.2 Method 2: [Cell density adjustment only]

In this method, cell density was modified according to the percentage of adenocarcinoma found in each biopsy core, assuming that the proportion of cancerous cells in each core reflects the total percentage of tumor cells within that segment. For segments with no detected tumor cells, a minimum value of 1% was assigned to account for potential undetected tumor sites. The newly generated single α/β ratio and log‐normal distribution of α were used in the TCP equations.

#### 2.6.3 Method 3: [Cell density + GP‐dependent α adjustment]

In addition to cell density adjustments, this method used discrete Gleason Pattern (GP)‐dependent alpha values for each segment instead of sampling α from a log‐normal distribution. Based on the methodology of Ghobadi et al.,^22^ it was assumed that the primary and secondary GPs comprised 75% and 25% of the tumor cell count, respectively.^7^ Therefore, *α*_GPi​_ values were applied in a 75:25 ratio within each voxel to match the segment's Gleason Score. The single α/β ratio was applied across all segments, and the alpha value corresponding to the lowest Gleason Pattern (*α*_GP2_) was assigned to segments where no cancer cells were detected.

#### 2.6.4 Method 4: [Cell density + GS‐dependent α/β ratio]

This method incorporated both cell density adjustments and GS‐dependent α/β ratios for each segment, in accordance with biopsy report. Here, *α* was sampled from the same log‐normal distribution range as in earlier methods. The α/β ratio associated with the lowest Gleason Score (α/β_GS2+2_) was assigned to segments where cancer cells were not identified.

### 2.7 Data analysis

Zhao et al.^7^ previously computed four Gleason Pattern (GP)‐dependent alpha parameters (*α*_GPi_​) for 20 different α/β ratios (detailed in Table 2 of their study). To explore the outcomes observed with Method 3, ten α/β ratios along with their corresponding *α*_GPi_​​ values from this list were randomly selected and a sensitivity analysis was conducted for each set. This analysis involved fixed TCP parameters to evaluate how the TCP varied with four *α*_GPi_ values from different sets​.

Later we focused on Methods 1, 2, and 4, comparing their effects on overall TCP calculations and TCP maps. To assess the statistical significance of overall TCP reduction from Method 1, a Wilcoxon signed‐rank test was performed, comparing the changes in overall TCP for Methods 2 and 4 relative to Method 1.

To compare voxel‐level TCP distributions between the relapsed GTV and nonGTV regions within the prostate, histograms of voxel‐level TCP values were generated for each patient and each method to visualize distribution differences across the TCP maps. The voxel‐level histograms were used for descriptive visualization only, without performing voxel‐wise statistical testing; therefore, no associated multiple comparison correction was required. Additionally, a volume‐weighted TCP calculation was performed on the TCP map to account for the volume‐dependent nature of overall TCP. The overall TCP of the nonGTV region was adjusted using a power‐root transformation based on its volume ratio relative to the GTV region, ensuring comparability. A Wilcoxon Signed‐Rank test was then conducted to evaluate the statistical significance of overall TCP differences between the relapsed GTV and nonGTV regions.

## 3 RESULTS

### 3.1 Radiosensitivity parameter generation

The numerically estimated Gleason‐Grade independent single α/β ratio derived from the secondary data was 1.77 Gy. An overall discrepancy of 2.3% was determined between the TCP values obtained from computed radiosensitivity parameters and the reported 5‐year biochemical or clinical failure‐free survival rates, encompassing outcomes from all three dose prescriptions in the CHHiP trial.^20^ The generated four GP‐dependent *α*_GPi_ values for the single α/β ratio 1.77 Gy and nine GS‐dependent α/β ratios (α/β_GSj_) for a log‐normal distribution of *α* within [0.05,0.40] Gy^−1^ are listed in Table 1 and Table 2 respectively:

**TABLE 1:** Generated four GP‐dependent alpha parameters (α_GPi_​) for the single α/β ratio of 1.77 Gy.

Single α/β (Gy)	Error (single α/β)	*α*_GP2_ (Gy^−1^)	*α*_GP3_ (Gy^−1^)	*α*_GP4_ (Gy^−1^)	*α*_GP5_ (Gy^−1^)	Error (*α*_GPi_)
1.77	2.27%	0.194	0.128	0.124	0.102	2.11%

**TABLE 2:** Generated nine GS‐dependent α/β ratios (α/*β*_GSj_) for log‐normal distribution of α.

α/β_GS2+2_ (Gy)	α/β_GS3+2_ (Gy)	α/β_GS3+3_ (Gy)	α/β_GS3+4_ (Gy)	α/β_GS4+3_ (Gy)	α/β_GS4+4_ (Gy)	α/β_GS4+5_ (Gy)	α/β_GS5+4_ (Gy)	α/β_GS5+5_ (Gy)	Error
1.54	1.87	2.05	2.11	2.16	2.34	2.67	2.92	3.05	1.04%

### 3.2 Overall TCP

The overall TCP values for the entire prostate calculated for the nine patients ranged from 86.1% to 96.5% using Method 1, which involved no parameter adjustments. In Method 2, the TCP decreased (relative to Method 1) for five patients, while it increased for the remaining four. Method 3 produced unrealistically high TCP values, nearing 100% for seven patients and increased TCP in all cases. In contrast, Method 4 notably reduced the overall TCP across all patients. Table 3 presents the calculated overall TCP values for each of the four methods for all nine patients.

**TABLE 3:** Overall TCP for nine patients across all four methods.

		Overall TCP^b^ of whole prostate (%)			
Patient ID	Risk group^a^	Method 1	Method 2	Method 3	Method 4
1	High	93.6	92.9	99.6	86.6
2	Intermediate	95.9	95.8	99.9	92.3
3	Intermediate	96.5	97.1	99.9	94.9
4	Intermediate	96.1	91.4	99.5	85.1
5	High	92.9	93.5	99.7	86.7
6	Intermediate	90.7	94.1	99.6	89.1
7	Intermediate	91.2	92.8	99.5	87.9
8	Intermediate	86.1	81.6	92.4	71.8
9	Intermediate	89.1	86.7	96.4	76.7

^a^ Risk group defined by the European Association of Urology (EAU) risk classification^26^.

^b^ TCP‐ tumor control probability.

### 3.3 TCP Maps

**FIGURE 3:**
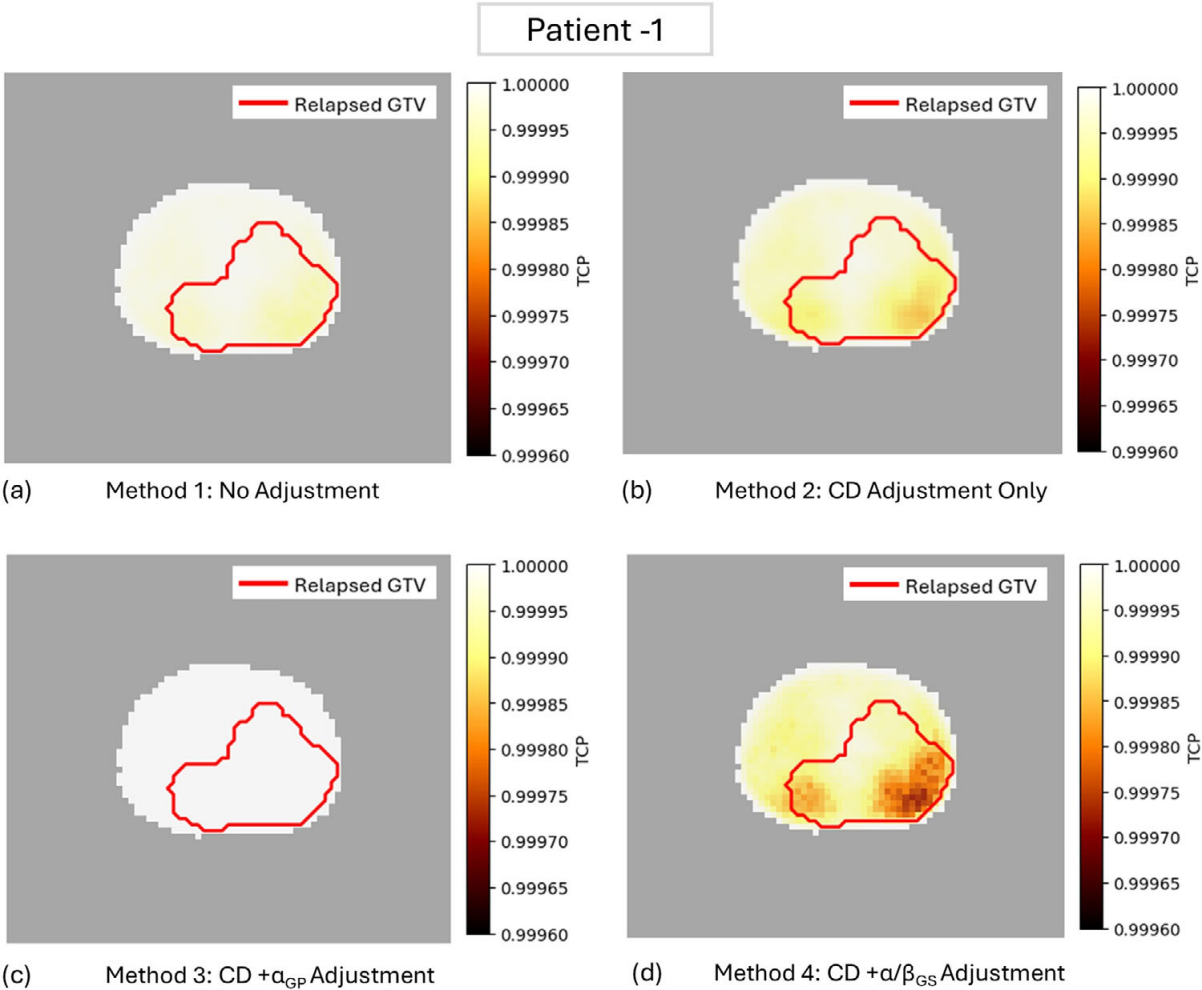
Axial views of TCP maps generated for Patient‐1 using (a) Method 1: No parameter adjustment, (b) Method 2: Cell density adjustment only, (c) Method 3: Cell density + GP‐dependent α adjustment, and (d) Method 4: Cell density + GS‐dependent α/β ratio. The darker regions indicate lower TCP values and the red contour line delineates the relapsed Gross Tumor Volume (GTV). Each subfigure is accompanied by a color bar (right) indicating voxel‐level TCP values.

The lower TCP regions were more prominently visible on the maps generated using Method 4 compared to Methods 1 and 2, where cell density and α/β ratios were adjusted segment‐wise based on biopsy reports. In contrast, Method 3, which incorporated cell density and GP‐dependent α adjustments, produced uniform maps with no distinct lower TCP regions suggesting this method does not have sufficient sensitivity to identify regions at risk of recurrence. Additionally, for seven out of nine patients (78%), the lower TCP regions in Method 4 maps closely aligned with the recurrent tumor regions (relapsed GTV). Figure 3 illustrates the TCP maps generated using all four methods for Patient 1.

### 3.4 Sensitivity analysis of 𝛼_GPi_ and statistical analysis

**FIGURE 4:**
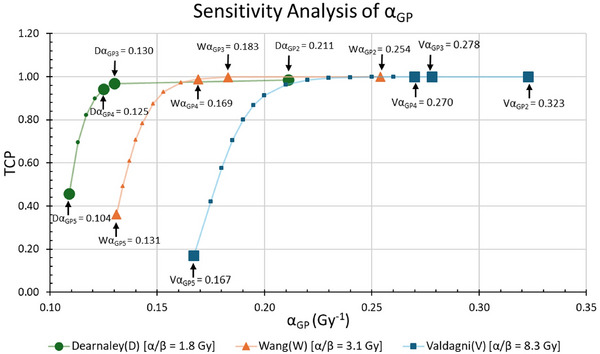
Sensitivity Analysis: TCP reduction corresponding to *α*_GP2_, *α*_GP3_, *α*_GP4,_ and *α*_GP5_ values computed by Zhao et al.^7^ for α/β ratios: 1.8 Gy (Dearnaley et al.^20^), 3.1 Gy (Wang et al.^19^) and 8.3 Gy (Valdagni et al.^27^).

Figure 4 illustrates the sensitivity analysis of α_GP2_, α_GP3_ ​, α_GP4,_ and α_GP5_, as computed by Zhao et al.,^7^ for three different α/β ratios: 1.8 Gy from Dearnaley et al.,^20^ 3.1 Gy from Wang et al.^19^ and 8.3 Gy from Valdagni et al.^27^ In all three cases, TCP variation remains minimal for *α*_GP2_, *α*_GP3_ ​, and *α*_GP4_ ​, whereas a pronounced drop in TCP is observed for *α*_GP5_​. These findings indicate that the TCP model is considerably more sensitive to the highest Gleason Pattern or *α*_GP5_.

According to the overall TCP calculations for the whole prostate in Table 3, Method 2 resulted in a TCP reduction in five out of nine patients or 56% of cases compared to Method 1, whereas Method 4 consistently reduced TCP for all patients (100%). The Wilcoxon signed‐rank test showed a statistically significant reduction in overall TCP for Method 4 compared to Method 1 (*p* = 0.004), whereas the reduction observed with Method 2 was not statistically significant (*p* = 0.570). Here, a lower overall TCP aligns with expectations, as all patients in the study experienced cancer recurrence.

**FIGURE 5:**
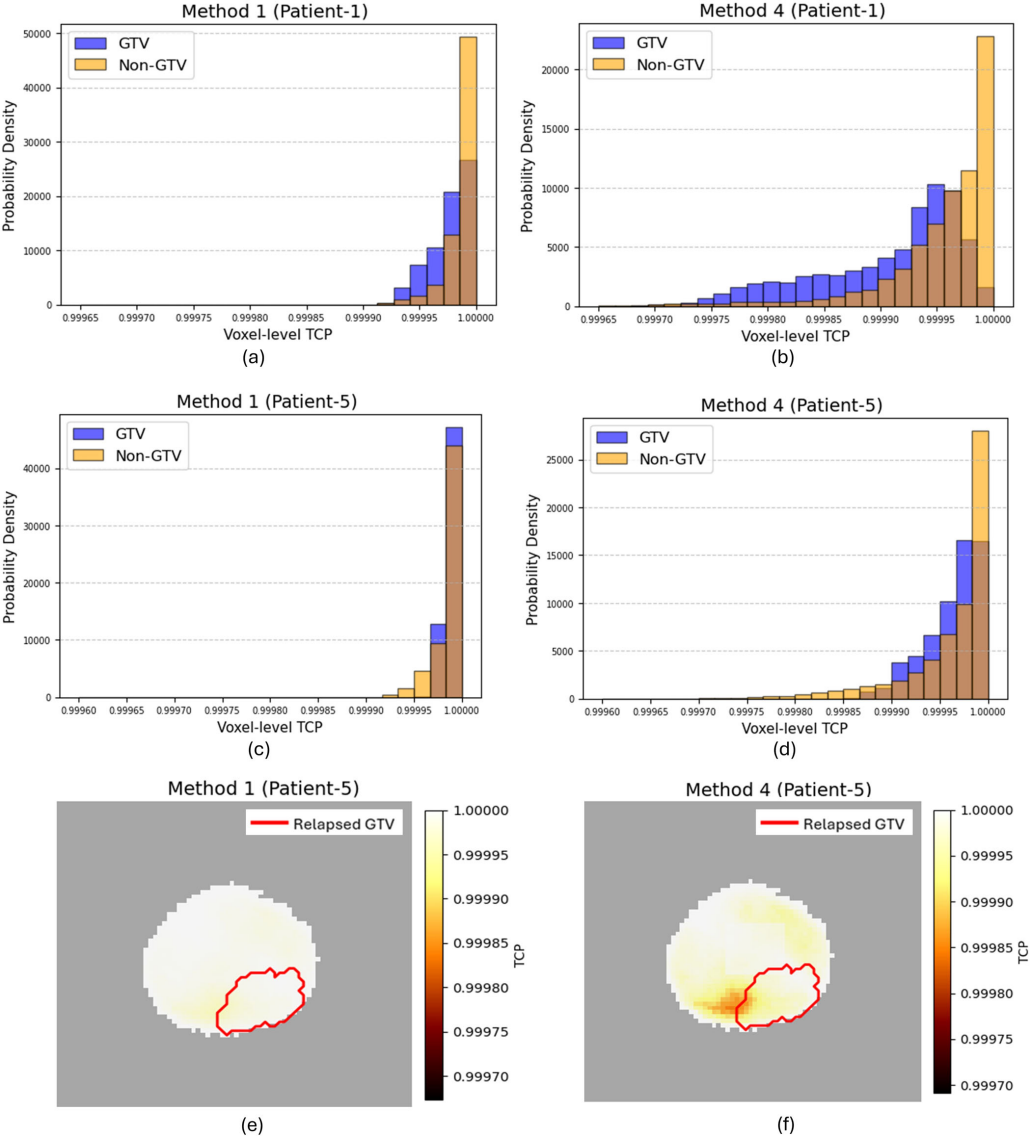
Histograms of voxel‐level TCP values for Patient‐1 using (a) Method 1 and (b) Method 4, corresponding to the TCP maps in Figure 3a,d, respectively. Similarly, histograms for Patient‐5 are shown for (c) Method 1 and (d) Method 4, with the corresponding TCP maps displayed in (e) and (f), respectively.

Visually identified regions of lower TCP values in the maps aligned well with the tumor recurrence location (relapsed GTV) in seven out of nine patients most visibly for Method 4. However, this spatial agreement was not as evident for the remaining two patients—Patient‐5 [Figure 5e,f] and Patient‐6. Figure 5a,b illustrates the voxel‐level TCP distributions from the maps derived by Method 1 and Method 4 for Patient‐1, whose lower TCP region showed strong spatial alignment with the relapsed GTV in the TCP maps [Figure 3a,d]. The histograms clearly indicate that lower TCP values are more concentrated within the GTV region for both methods, with Method 4 demonstrating an improved distribution by further reducing and spreading lower TCP values within the GTV compared to Method 1. Conversely, Figure 5c,d display the voxel‐level TCP distributions from Method 1 and Method 4 respectively for Patient‐5, whose lower TCP region did not align well with the relapsed GTV. In both methods, lower TCP values were more prevalent in the nonGTV region than in the GTV region. However, despite the misalignment in spatial localization for recurrence prediction, Method 4 still demonstrated an improvement by further reducing voxel‐level TCPs within the GTV compared to Method 1.

**FIGURE 6:**
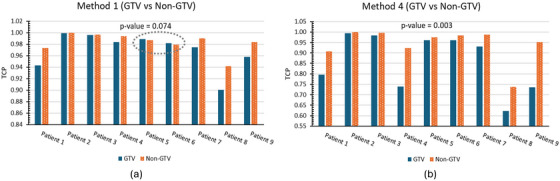
Volume‐weighted TCP comparisons between the relapsed GTV and nonGTV regions for each of the nine patients for (a): Method 1 and (b): Method 4. The dotted circle in (a) highlights a relatively lower volume‐weighted TCP in the nonGTV region compared to the GTV for Patient‐5 and Patient‐6 in Method 1.

The volume‐weighted overall TCP calculations further support these observations. For all patients except Patient‐5 and Patient‐6, overall TCP was lower in the relapsed GTV region compared to the nonGTV region when no parameter adjustment was applied in Method 1 [Figure 6a] (*p* = 0.074). Both Method 2 and Method 4 improved this alignment, reducing the overall TCP in the GTV for all patients. However, the difference in volume‐weighted overall TCP between GTV and nonGTV regions was statistically more significant in Method 4 (*p* = 0.003) than in Method 2 (*p* = 0.012), reinforcing the effectiveness of Method 4 in refining TCP predictions.

## 4 DISCUSSION

Prostate cancer is commonly a multifocal disease^28^ and exhibits considerable histopathological heterogeneity.^22^ Tumors with higher GS are often assumed to be associated with greater radioresistance and increased risk of treatment failure.^22^ This underscores the importance of selecting accurate radiosensitivity parameters to estimate radiation response reliably.^29^ Our study demonstrated that adjusting the α/β ratio based on tumor grading derived from histopathology analysis of biopsy samples, combined with cell density adjustment, has the greatest impact on enhancing the PCa recurrence prediction capability of our atlas‐based TCP model. Given prostate cancer's heterogeneity, Lennernas et al.^30^ suggested the plausibility of varying radiosensitivity within tumor sub‐volumes, with some regions resembling radiosensitivity of other cancers. They cautioned against assuming a uniformly low α/β ratio for prostate cancer, as even a small subset of cells with higher ratios can dramatically influence overall treatment response. A systematic review by Leeuwen et al.^29^ emphasized that tumor site and histology independently influence radiosensitivity and strongly recommended reporting α/β values separately for each different histology. They advocated using a range of possible α/β values rather than a single estimate in clinical practice, which is strongly supported by the outcomes of our study. Ghobadi et al.^22^ demonstrated that considering radioresistance increases with GS broadens dose–response curves, aligning with clinical observations. They further suggested that a higher dose differentiation between different tumor volumes might be feasible without compromising TCP, when the relationship between radiosensitivity and GS for each focal lesion is considered.

Ebert et al.'s^31^ study demonstrated that optimizing a dose distribution to maximize TCP without dose escalation requires an understanding of the clonogenic cell density and their radiosensitivity characteristics within the tumor. However, Bentzen et at^32^ found that heterogeneity in radiosensitivity has a greater impact on dose–response curves than cell density heterogeneity alone. In line with these studies, our findings also demonstrated that combining cell density and α/β adjustments (Method 4) led to a more pronounced TCP reduction than cell density adjustments alone (Method 2). On the other hand, Lennernas et al.^30^ highlighted the importance of conducting sensitivity analyses with varying α/β ratios and total doses before implementing new fractionation schedules in clinical practice. Supporting these recommendations, our sensitivity analysis of α/β parameters (Section 2.5.3) also reinforces the importance of histology‐dependent α/β values for reliable TCP estimation for effective prediction of recurrence.

Our GS‐dependent α/β values exhibited an increasing trend with higher Gleason Scores, yet all nine α/β values remained below 3.1 Gy,^19^ consistent with the widely recognized lower α/β ratios in prostate cancer.^23^, ^24^, ^25^ During the generation of GS‐dependent α/β values, we observed greater sensitivity of α/β to the lower boundary than the upper boundary. Consequently, we reduced the lower boundary to 1 Gy and selected 8.3 Gy as the upper boundary.^7^, ^20^ Our minimum α/β value (α/β_GS2+2_​) of 1.54 Gy and the single α/β ratio of 1.77 Gy align closely with retrospective reviews conducted by Dearnaley et al.,^20^ which analyzed large patient databases and suggested that the best estimates for the α/β ratio range between 1.4 and 1.9 Gy, though values as high as 8.3 Gy have been reported. Additionally, the CHHiP trial estimated the α/β ratio for prostate cancer to be 1.8 Gy, which is in very close agreement with our result of 1.77 Gy for the single α/β. The four GP‐dependent *α* values generated for α/β = 1.77 Gy also closely correspond to those generated by Zhao et al.^7^ for Dearnaley's α/β = 1.8 Gy. However, Zhao et al. raised concerns about the derivation of α_GPi_​ values, particularly *α*_GP2_​, suggesting that it may have had a limited impact on the optimization function, likely due to the low proportion of GP2 tumor cells within the cohort.^7^ Our sensitivity analysis [Figure 4] demonstrated that TCP values are highly sensitive to *α*_GP5_ ​, further supporting the suggestion that poorly differentiated cancer cells are more radioresistant.^22^ Interestingly, our cohort did not include any cases of GP5, so no noticeable reduction in TCP was observed when applying GP‐dependent *α* values in Method 3. Additionally, the total tumor cell number for GS 5+5 found in the secondary dataset was nearly zero, raising concerns about the accuracy of the α/β_GS5+5​_ value.

Haworth et al.^17^ previously demonstrated the radiobiological model's ability to predict biochemical control. The study involved dividing the prostate into 12 sub segments and initially assigning clonogen numbers to each segment based on tumor probability data from Zeng et al.^33^ The model's predictive power was later enhanced by modifying tumor cell densities using patient specific biopsy reports. This adjustment aimed to lower TCP values in high‐risk regions while maintaining higher TCP values in low‐risk areas of equivalent underdosing. The study highlighted the potential of customizing model parameters for high‐risk features, such as aggressive tumors or hypoxia. In contrast, our study incorporated population‐based atlases as well as patient‐specific biopsy report–informed data and radiosensitivity parameter adjustments, which showed improved performance in recurrence prediction.

Zhao et al.^12^ previously utilized the CD‐atlas and TP‐atlas to generate prescription doses based on TCP distributions for cases where patient‐specific information was unavailable. The TP‐atlas played a crucial role in a qualitative aspect by identifying potential tumor regions. In its absence, TCP maps tended to exhibit uniform distributions, failing to predict possible relapse locations effectively. However, a limitation of the TP‐atlas is that it was developed using axial histology sections, excluding the most superior and inferior portions of the prostate base and apex, which were sectioned sagittally as per clinical protocols.^12^, ^16^ Since tumors are predominantly found in the peripheral zone (PZ),^12^, ^22^ as expected, the population‐based atlas shows a higher probability of identifying tumors near the PZ and a lower likelihood in the non‐PZ areas. Therefore, the predictive power of the atlas alone may be reduced for tumors located in the base, apex, and non‐PZ regions, highlighting the need to incorporate patient‐specific biopsy information where possible. It is also important to consider that as a population‐based model, the atlas may not fully represent the tumor distribution patterns of individual patients within this cohort.

Some other limitations of this study include the small number of patients, all of whom were drawn from a focal SBRT re‐irradiation trial for biopsy‐proven local recurrence. In addition, the availability of data from their primary prostate cancer treatment was limited; as such, caution should be exercised in extrapolating these findings to the broader prostate cancer population treated with initial definitive radiotherapy. Diagnostic images prior to the primary treatment were largely unavailable, and in the few accessible cases, the GTV was generally not delineated —understandably so, given the clinical practices at the time. Consequently, the histopathology report was the sole source of information regarding the primary tumor's characteristics and location. Histopathology, however, has its own limitations, including the potential for missing tumor sites during sampling. Furthermore, each patient's prostate was segmented differently into anatomical regions, requiring a geometrical division of individual prostate atlases based on estimated anatomy to align with the biopsy data. This approach may have resulted in missed or overestimated potential tumor sites. Additionally, TCP modeling was performed at the voxel level, but intervoxel dependencies were not accounted for, which may affect the spatial coherence of the predicted distributions. Segment‐wise parameter modifications led to maps that occasionally lacked smoothness of TCP distribution. Another potential source of uncertainty lies in the deformable registration of the atlases to individual patient prostate contours. Although internal volumetric accuracy could not be assessed due to the lack of consistent internal anatomical landmarks (e.g., peripheral zone or urethra contours), the quantitative evaluation using the mean absolute surface distance (MASD) showed consistently small errors (<1.5 mm across all patients), suggesting that residual registration inaccuracies are unlikely to have materially influenced the results. Given these limitations and the small, highly selected cohort, our findings should be regarded as proof‐of‐principle, demonstrating feasibility rather than generalizability at this stage. Nevertheless, the model demonstrated promising capability in predicting the location of tumor recurrence. Clinically, it may be incorporated into treatment planning through biological optimization approaches,^9^ with structure‐based methods^14^ providing an interim pathway until commercial treatment planning systems support full biological modelling.

## 5 CONCLUSION

This study demonstrated the potential of an atlas‐based TCP model to predict prostate cancer recurrence by integrating patient‐specific histopathology data. Gleason Grade‐dependent radiosensitivity parameters were derived and incorporated into the TCP model, enabling the calculation of overall TCP and the generation of patient‐specific TCP distribution maps using different segment‐wise parameter adjustment methods. Adjusting both cell densities and α/β parameters based on histopathology resulted in lower overall TCP values for all nine patients and alignment of lower TCP regions with relapsed tumor sites in seven patients. These findings highlight the superior effectiveness of this approach in predicting the location of a recurrence compared to other parameter adjustment methods, such as cell density alone, cell density combined with α parameter adjustments, or no parameter adjustment at all.

The model offers potential for generating personalized, optimized dose prescriptions through voxel‐level TCP‐based dose redistribution, which could improve overall TCP prediction and effectively reduce recurrence risk. Future studies should validate this model in larger patient cohorts, including external validation to confirm the robustness and generalizability of results, and explore the integration of advanced tools such as autosegmentation, radiomics features, and additional prognostic factors (e.g., PSA dynamics, T‐stage, age, and the impact of androgen deprivation therapy) to further enhance its predictive accuracy.

## CONFLICT OF INTEREST STATEMENT

The authors declare no conflicts of interest.

## Supporting information



Supporting information
