# Supplementary material for: Predicting prostate cancer recurrence using an atlas‐based tumor control probability model
Source: Med Phys. 2026 Jan 14;53(1):e70282. doi: 10.1002/mp.70282 (PMC12801181; doi:10.1002/mp.70282)
Supplement: Supplementary file 1 — Supporting information [file MP-53-0-s001.docx]

**Supporting Information**

**Predicting Prostate Cancer Recurrence Using an Atlas-Based Tumor Control Probability Model**

1. **Patient demographics and treatment summary**

**Table S-1:** Primary prostate cancer and treatment related data based on selection criteria for nine patients

| Patient ID | Primary Prostate Cancer & Treatment Data | | | | | |
| --- | --- | --- | --- | --- | --- | --- |
|  | Risk Group^a^ | Overall Gleason Score | Dose Prescription (Total dose/Fraction) | Treatment  Duration  T_exp_  (days) | Biopsy Report | DICOM^b^ Planning Data |
| 1 | High | 4+4 | 80 Gy/40 | 62 | ✓ | ✓ |
| 2 | Intermediate | 4+3 | 80 Gy/40 | 61 | ✓ | ✓ |
| 3 | Intermediate | 3+4 | 83.2 Gy/40 | 61 | ✓ | ✓ |
| 4 | Intermediate | 3+4 | 81.6 Gy/40 | 61 | ✓ | ✓ |
| 5 | High | 4+4 | 80 Gy/40 | 62 | ✓ | ✓ |
| 6 | Intermediate | 3+3 | 78 Gy/39 | 57 | ✓ | X |
| 7 | Intermediate | 3+4 | 81 Gy/45 | 66 | ✓ | X |
| 8 | Intermediate | 3+4 | 70 Gy/35 | 47 | ✓ | X |
| 9 | Intermediate | 4+3 | 73 Gy/37 | 52 | ✓ | X |

^a^Risk Group defined by the European Association of Urology (EAU) risk classification^1^

^b^DICOM:  Digital Imaging and Communications in Medicine

1. **TCP model**

In the voxel-wise TCP model, tumor control probability is calculated individually for each voxel, using inputs such as the tumor cell count (Ni), radiosensitivity parameters α and α/β ratio, the dose per fraction (d_i_), total number of fractions (n), overall treatment duration (T_exp_), and the potential doubling time (T_pot_).

The cell density atlas (CD-atlas) includes both tumor and normal cells, so estimating the number of tumor cells specifically was necessary. For a voxel i classified as tumor, the tumor cell count N_i_ ​ can be estimated using:

$N_{i}= A_{risk}. \rho_{i}. V (1)$

where the product of cell density ρ_i_ and voxel volume V yields the total cell count (encompassing both normal and tumor cells) within voxel i. A scaling factor A_risk_​ was applied to adjust the total cell count in proportion to the median tumor cell numbers for each risk group, as estimated by Wang et al. This normalization factor, A_risk_​, essentially represents the fraction of cells in a voxel that are cancer cells.

**Parameter definitions:**

**α (alpha):** The linear radiosensitivity parameter, representing the probability of lethal damage per unit dose.

**β (beta):** The quadratic radiosensitivity parameter, representing the component of cell killing proportional to the square of dose, associated with two-hit damage.

**α/β ratio:** The dose at which the contributions of the linear and quadratic components are equal; it reflects a tissue or tumor’s sensitivity to fraction size.

**T_pot_ :** The theoretical time required for the tumor cell population to double in the absence of cell loss.

**2.1 Overall TCP calculation**

The TCP in the i^th^ voxel is calculated following a sampling method from a log-normal distribution of cell density from the CD-atlas and a log-normal distribution of α_k_ from the range of [0.05, 0.40] with a mean ᾱ=0.15 Gy^−1^and standard deviation σ_α_=0.04 Gy^−1 2–5^.

The equation is given below:

$$TCP_{i}\left( N_{i,M},\alpha_{k}, d_{i} \right)=\exp[-N_{i,M} exp(-\alpha_{k}nd_{i}-\frac{\alpha_{k}nd_{i}^{2}}{\frac{\alpha}{\beta}}+\ln(2)\frac{T_{exp}}{T_{pot}})] \alpha_{k}\epsilon[0.05, 0.4] (2)$$

The M^th^ sample in the i^th^ voxel, N_i,M_ was randomly sampled (from total U samples) assuming a log-normal cell distribution using the pre-registered ‘mean CD-atlas’ and ‘standard deviation CD-atlas’. Then using the weighting factor ω(N_i,M_) derived from the log-normal distribution’s probability density at the voxel level, and summing over the U sampled values of N_i,M_, the overall TCP for voxel i is computed as:

$TCP_{i}\left( N_{i,M},\alpha_{k} \right)=\frac{1}{\sum\omega(N_{i,M})}\sum_{M=1}^{U} \omega\left( N_{i,M} \right)\mathrm{TCP}_{i}\left( N_{i,M},\alpha_{k}, d_{i} \right) (3)$

Thus, the TCP for the entire prostate for k^th^ α​ sample is given by:

$TCP\left( \alpha_{k} \right)=\prod_{i=1}^{M} \mathrm{TCP}_{i} \left( N_{i,M}, \alpha_{k} \right) \alpha_{k}\epsilon[0.05, 0.4] (4)$

Finally, the overall TCP was calculated by taking the weighted average from entire prostate TCP values of each α_k_ and the corresponding weighting factor ω(α_k_) derived from the normalized probability density of the log-normal distribution:

$TCP=\frac{1}{W} \sum_{k=1}^{H} \omega(\alpha_{k})TCP\left( \alpha_{k} \right) \alpha_{k}\epsilon[0.05, 0.4] (5)$

The potential doubling time T_pot_ was set to 42 days, following the values reported by Wang et al.^5^ Dose per fraction d_i_, total fraction n, and treatment duration T_exp_ were derived from each patient’s primary treatment dose schedule.

**2.2 TCP Map Generation**

To generate TCP maps, the mean tumor probability atlas (TP-atlas) was used in conjunction with the CD-atlas, following the methodology outlined by Zhao et al.^4^ The TP-atlas indicates the mean probability of finding tumor cells in each voxel. Therefore, equation 2 was updated as shown in Equation 6, followed by the subsequent equations:

$${TCP}_{i}\left( N_{i,M},\alpha_{k},d_{i} \right)=\exp[-N_{i,M} exp(-\alpha_{k}nd_{i}-\frac{\alpha_{k}nd_{i}^{2}}{\frac{\alpha}{\beta}}+\ln(2)\frac{T_{exp}}{T_{pot}})] .{TP}_{i}+\left( 1-{TP}_{i} \right) \alpha_{k}\epsilon\left[ 0.05, 0.4 \right] (6)$$

In contrast to our previous work that estimated the overall TCP for the whole prostate by step-wise calculating the TCP for each alpha value (equation 4), this work calculates the TCP at the voxel level using Equations 3 and 5 to derive the overall TCP.

1. **Sensitivity Analysis: TCP Vs α/β ratios**


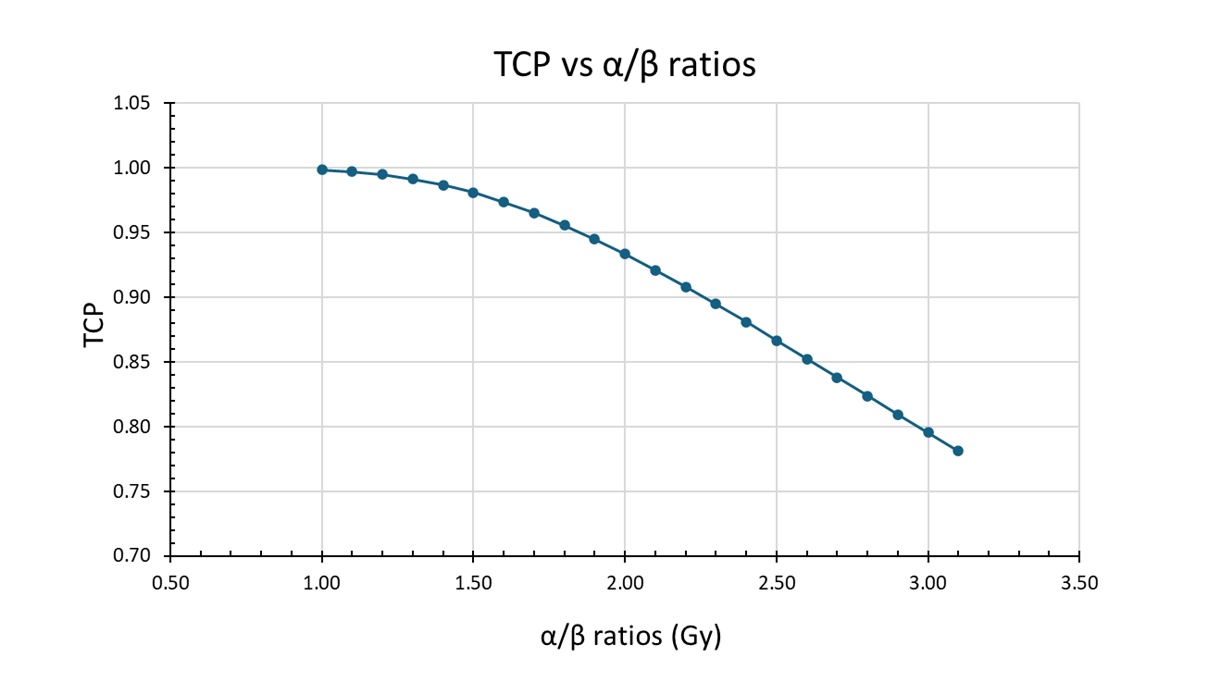


**Figure S-1:** Sensitivity analysis of the relationship between tumor control probability (TCP) and α/β ratios, performed with fixed parameters (Ni, di, n, etc.). The analysis demonstrates a consistent decrease in TCP with increasing α/β, showing a nearly linear trend beyond α/β = 1.5 Gy.

**References:**

1. Prostate Cancer - Uroweb. Accessed January 5, 2025. https://uroweb.org/guidelines/prostate-cancer/chapter/classification-and-staging-systems

2. Zhao Y, Haworth A, Reynolds HM, et al. Patient‐specific voxel‐level dose prescription for prostate cancer radiotherapy considering tumor cell density and grade distribution. *Med Phys*. 2023;50(6):3746-3761. doi:10.1002/mp.16264

3. Her EJ, Haworth A, Reynolds HM, et al. Voxel-level biological optimisation of prostate IMRT using patient-specific tumour location and clonogen density derived from mpMRI. *Radiat Oncol*. 2020;15(1):172. doi:10.1186/s13014-020-01568-6

4. Zhao Y, Haworth A, Reynolds HM, et al. Towards optimal heterogeneous prostate radiotherapy dose prescriptions based on patient‐specific or population‐based biological features. *Med Phys*. 2024;51(5):3766-3781. doi:10.1002/mp.16936

5. Wang JZ, Guerrero M, Li XA. How low is the/ratio for prostate cancer. *Int J Radiat Oncol Biol Phys*. 2003;55(1):194-203.
